# All‐PEG‐Like Block Copolymers Self‐Assemble into Stealth Nanocarriers for Drug Delivery

**DOI:** 10.1002/advs.202517048

**Published:** 2026-01-18

**Authors:** Parul Sirohi, Brooke E. Silverstein, Yulia Shmidov, Sonal Deshpande, Joy Tong, Yvonne Y. Ma, Chinmay S. Potnis, Soumen Saha, Xinghai Li, Max R. Ney, Sarah Y. Kim, Joshua J. Milligan, Lixin Fan, Matthew L. Becker, Daniel Reker, Ashutosh Chilkoti

**Affiliations:** ^1^ Department of Biomedical Engineering Duke University Durham North Carolina USA; ^2^ Basic Science Program, Frederick National Laboratory for Cancer Research SAXS Facility of the National Cancer Institute Frederick Maryland USA; ^3^ Department of Chemistry Duke University Durham North Carolina USA; ^4^ Thomas Lord Department of Mechanical Engineering and Material Science Duke University Durham North Carolina USA

**Keywords:** anti‐PEG, block copolymer, drug delivery, stealth nanoparticles, self‐assembly

## Abstract

Poly(oligo(ethylene glycol) methyl ether methacrylate) (POEGMA) is a stealth polymer that does not exhibit polyethylene glycol (PEG) antigenicity. Herein, we engineered self‐assembling nanoparticles composed entirely of POEGMA by designing AB diblock copolymers with varying oligo(ethylene glycol) (EG) side chains. We found that a one‐unit difference between di‐ and tri‐ethylene glycol side chains is sufficient to induce amphiphilicity and enables temperature‐triggered self‐assembly above room temperature when the block length ratio is at least 0.25. To broaden the temperature stability window, we increased amphiphilicity by incorporating mono‐ethylene glycol into the hydrophobic block via random copolymerization, yielding nanoparticles stable between 20°C–40°C. These POEGMA nanoparticles effectively encapsulate diverse hydrophobic drugs with high loading efficiency. Notably, POEGMA‐encapsulated doxorubicin retained the drug's in vitro activity and exhibited enhanced in vivo efficacy compared to free doxorubicin due to improved pharmacokinetics. Furthermore, these nanoparticles demonstrated stealth behavior by evading recognition by anti‐PEG antibodies. This study introduces a versatile, fully POEGMA‐based platform for stealth drug delivery with tunable thermal responsiveness and high therapeutic potential.

## Graphical abstract

Self‐assembling nanoparticles composed entirely of POEGMA are engineered by exploiting side‐chain–dependent amphiphilicity and tunable thermoresponsive behavior. These single‐material nanocarriers encapsulate diverse hydrophobic drugs with high efficiency, retain therapeutic activity, improve in vivo efficacy, and evade anti‐PEG antibodies. The work establishes a versatile stealth alternative for next‐generation drug delivery.

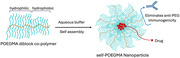

## 1 Main

Amphiphilic block copolymers can self‐assemble into nano‐ to meso‐scale structures [1, 2]. Polyethylene glycol (PEG) is a common hydrophilic block in block copolymers that self‐assemble into nanoparticles for biomedical applications [3]. PEG is a popular choice for the hydrophilic, corona‐forming block because the PEG corona provides “stealth” behavior that imparts a long plasma residence time to the nanoparticle [4, 5]. PEG is also non‐toxic and has a long history of relatively safe use in humans, as many approved biologic drugs are conjugated to PEG [4], and PEG is commonly used as an excipient in many drug formulations [6]. However, the ubiquity of PEG in consumer products, drug excipients, and vaccines has turned into a double‐edged sword, as it has led to the emergence of anti‐PEG antibodies in the past three decades. Recent reports indicate that more than 60% of the US population has significant levels of circulating IgG and IgM anti‐PEG antibodies [7, 8, 9].

This raises several concerns, including the potential opsonization of the PEG nanoparticles, leading to premature clearance through the reticuloendothelial system and the incidence of rare—but potentially fatal—anaphylactoid reactions to PEGylated nanoparticles [10, 11]. Unfortunately, this problem is becoming more serious, as the frequency of exposure to PEG is increasing because of repeated immunization of people with the COVID‐19 vaccines that contain PEG in their formulation [12, 13]. Beyond the immunogenicity concerns of PEG, the core‐forming block in these polymeric nanoparticles is composed of a more hydrophobic polymer such as polypropylene oxide (PPO), polystyrene oxide (PSO), polylactic acid (PAA), etc., some of which are poorly degradable or are potentially toxic [14, 15].

Building upon the long history of safe use of PEG in the human body, but recognizing that PEG has limitations related to its—increasing—immunogenicity and the potential limitations of hydrophobic monomers used for the core‐forming block in PEG‐decorated polymeric nanoparticles, we sought to create nanoparticles that are solely composed of PEG‐like moieties, but that lack the immunogenicity of PEG. To achieve this goal, we build upon our previous work on the development of a next‐generation PEG‐like stealth polymer poly(oligo ethylene glycol methyl ether methacrylate) (POEGMA) that presents short oligomers of ethylene glycol (EG)n as the side chain [16]. We have previously shown that when the EG side‐chain length in POEGMA —n— is ≤3, the polymer does not bind to pre‐existing human anti‐PEG antibodies, nor does it elicit a significant IgM or IgG response against itself in mice [16, 17, 18]. Additionally, POEGMA is a thermally responsive polymer with lower critical solution temperature (LCST) phase behavior, wherein POEGMA phase separates from an aqueous‐soluble phase into two immiscible phases above a critical transition temperature (*T*_t_) [19, 20]. We have previously reported that the *T*_t_ of POEGMA can be tuned by manipulating the side chain length of the monomers, where monomers with shorter EG units are relatively more hydrophobic and hence exhibit a lower *T*_t_ [20].

Here, we exploit these attributes of POEGMA to design nanoparticles that are composed solely of POEGMA that can self‐assemble into nanoparticles, and which can also coacervate into an insoluble micellar phase with an increase in temperature. We synthesized a library of simple AB‐type block copolymers with EG3MA and EG2MA as the monomer in the two blocks with varied relative degree of polymerization (DP) of each block. We then screened this EG3‐EG2 diblock copolymer library for self‐assembly as a function of temperature and found that a subset self‐assembled into spherical micelles, but the CMT of these polymers was above room temperature.

To further tune the CMT of the block copolymers, we also synthesized a slightly more complex EG3‐EG2 diblock POEGMA library where the hydrophobic block is a random copolymer of EG2 and EG1 to systematically tune the amphiphilicity of the two blocks. We show that this diblock POEGMA produces nanoparticles via self‐assembly into micelles, and the temperature range over which the micelles are stable can be tuned by the composition of the two blocks and span over physiologically relevant temperatures for biomedical applications. Finally, as proof‐of‐concept of their utility for biomedical applications, we demonstrate that a range of small‐molecule chemotherapeutics can be encapsulated in these nanoparticles with high efficiency and that the nanoparticles are pharmacologically active both in vitro and in vivo.

## 2 Design and Synthesis of Simple AB‐Type EG3‐EG2 Diblock POEGMA Library

**FIGURE 1:**
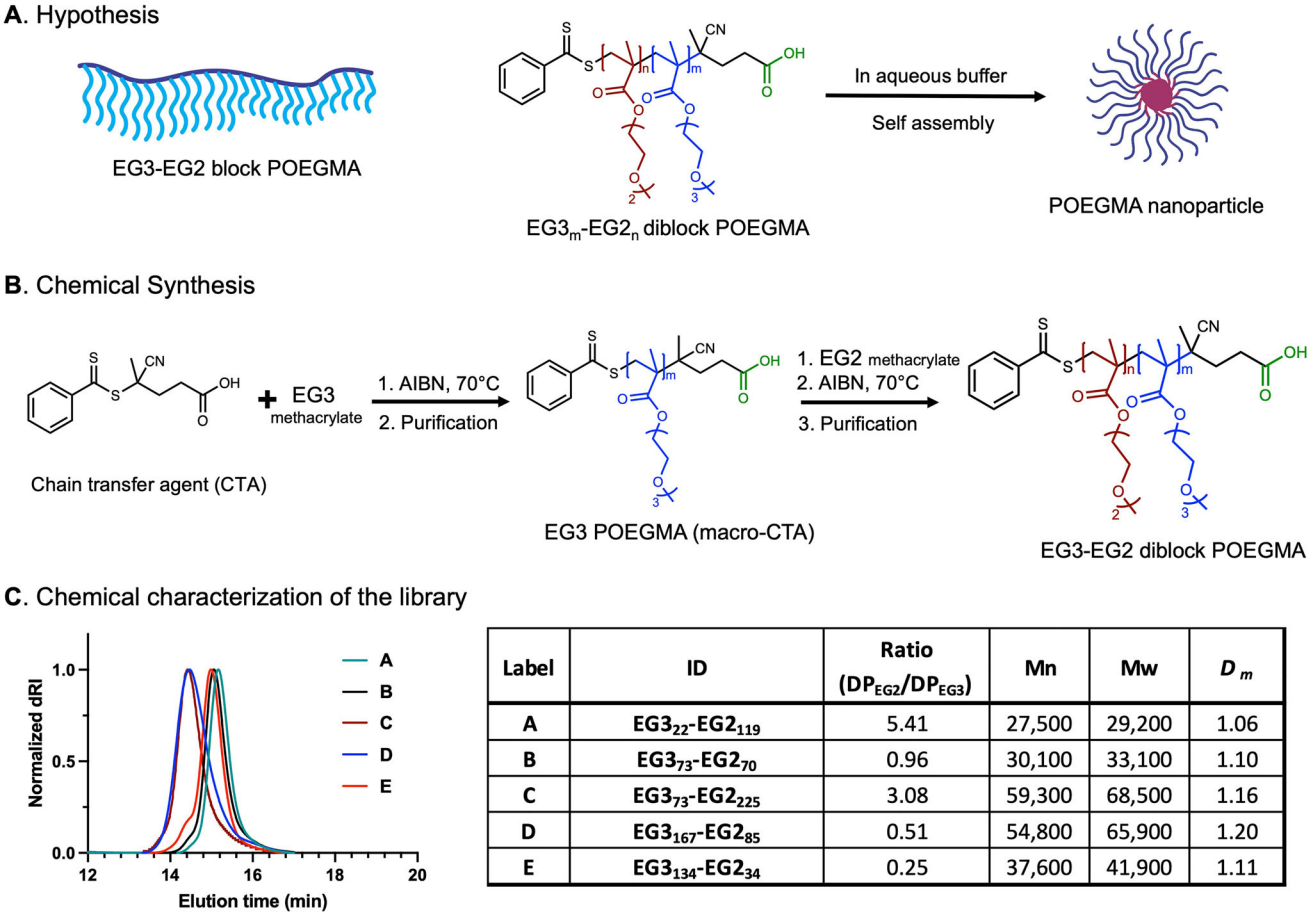
Design and synthesis of an AB‐type EG3‐EG2 diblock POEGMA library where each block is a homopolymer. (**A**) We hypothesize that POEGMA diblock copolymers composed of EG2 and EG3 side chains in the two blocks will self‐assemble into nano‐scale particles in aqueous media. (**B**) Two‐step chemical synthesis scheme of the diblock POEGMA via RAFT polymerization reaction. (**C**) Chemical characterization of the diblock library: SEC chromatograms and summary table of the diblock POEGMA library for M_n_, M_w_, and *D* *_m_* of the polymers determined by SEC‐MALS. The ID of the diblock POEGMA represents the degree of polymerization (DP_n_) of the individual blocks determined by M_n_ divided by the molecular masses of the respective monomer. Each polymer is given an alphabetical label to simplify the nomenclature.

We previously discovered that a shorter EG side chain, being less hydrophilic, decreases the *T*_t_ of the polymer and that a side chain length ≤ 3 is not immunogenic [16, 17, 18, 20]. Hence, EGnMA with n ≤ 3 were the monomers of choice for this study, under the reasonable assumption that nanoparticles composed of these monomers would also be non‐immunogenic. We hypothesized that a block copolymer where one block consists of repeats of EG3MA and the other of EG2MA would impart enough amphiphilicity to the diblock copolymer for its self‐assembly into nanoparticles with the EG3 block forming the corona and the EG2 block forming the core of the nanoparticles (Figure 1A).

To test this hypothesis, we synthesized a set of EG3‐EG2 diblock copolymers with a varied degree of polymerization (DP) of the EG2 monomer—as the hydrophobic block—and the EG3 monomer—as the hydrophilic block. The synthesis was accomplished in two steps of reversible addition‐fragmentation chain‐transfer (RAFT) polymerization (Figure 1B). The first RAFT reaction was quenched at 80%–90% monomer conversion to ensure an intact chain transfer agent (CTA)‐end group on the resulting EG3 POEGMA that could be employed as a macro‐CTA for the synthesis of the EG2 block by a subsequent round of RAFT. The resulting EG3 block and the final EG3‐EG2 diblock copolymers were characterized for % monomer conversion and apparent molecular mass by NMR. All the purified polymers, including the first EG3 block and the final diblock, were also characterized for number average molecular weight (M_n_), weight average molecular weight (M_w_), and molar mass distribution (*D_m_* = M_w_/M_n_) using size exclusion chromatography with an in‐line multi angle light scattering (SEC‐MALS) detector (Figure 1C). The DP of the individual block was determined by its M_n_ divided by the formal weight of the respective monomers. Only diblock polymers with a *D_m_* ≤ 1.2 were studied further to minimize the effect of mass distribution on their measured properties and self‐assembly behavior. Each diblock polymer in the set was given a unique ID that describes the DP of the individual blocks and an alphabetical label for easy identification (Figure 1C). The relative block lengths of each block in the diblock POEGMA library were varied with the goal of covering a wide range of amphiphilicities.

### 2.1 Physical Characterization of the Diblock POEGMA Library

**FIGURE 2:**
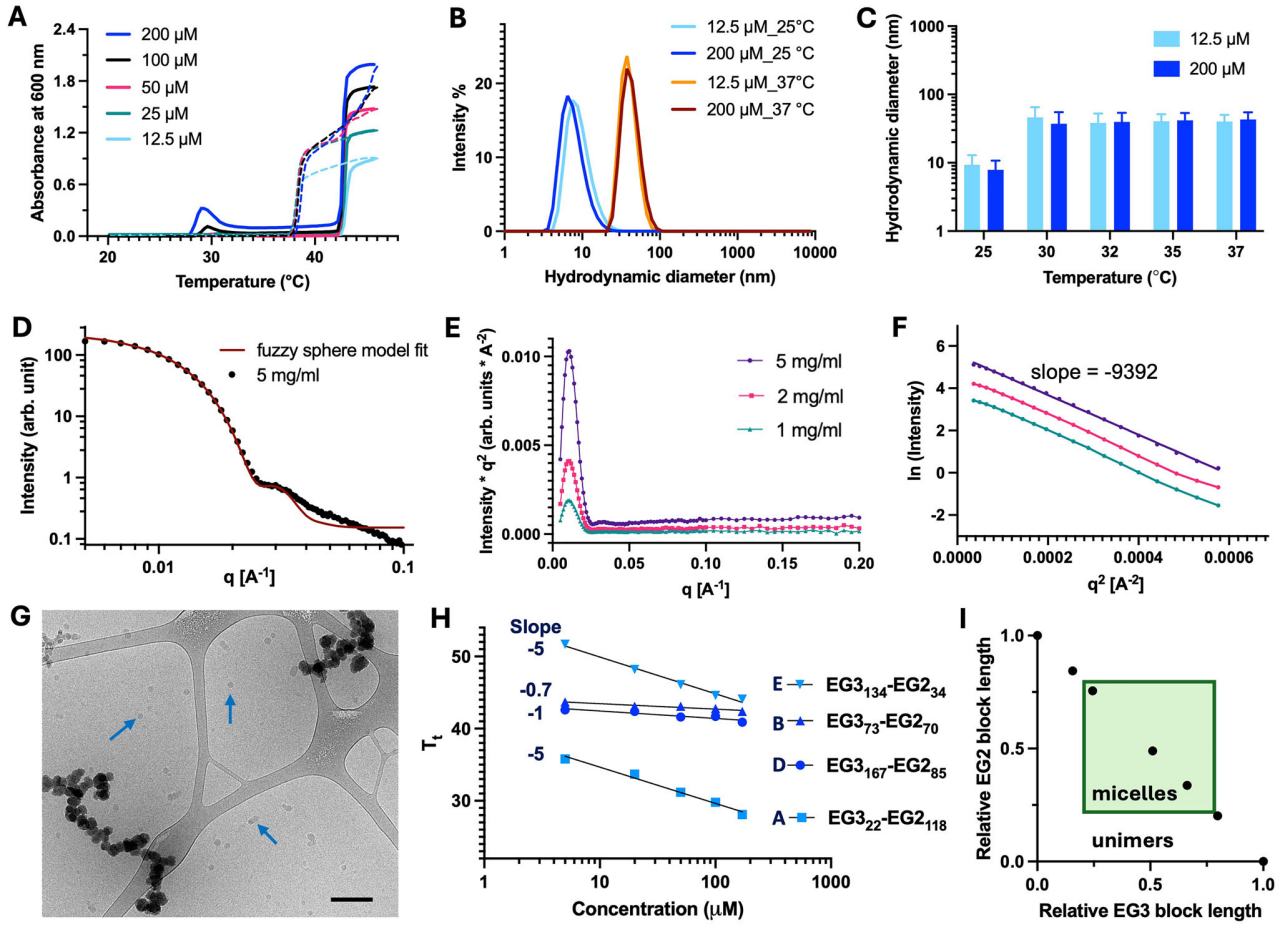
Physical characterization of polymer D: EG3_167_‐EG2_85_ (A–G), and summary of self‐assembly of diblock EG3‐EG2 POEGMA library (H‐I). (A) Absorbance at 600 nm of polymer D at various concentrations as a function of solution temperature. Heating curves are represented by solid lines, and cooling curves are represented by dashed lines. A sharp increase in absorbance at 42°C indicates the cloud point (*T*_t_) of this polymer. (B) Change in hydrodynamic diameter of polymer D from 25°C (below CMT) to 37°C (above CMT but below *T*_t_) at low (12.5 µM) and high concentration (200 µM). (C) Hydrodynamic diameter of polymer D for a range of biologically relevant temperatures below *T*_t_. The DLS measurements were recorded at least 3‐times and the data are presented as mean ± SEM. (D) 1D SAXS data at 37°C for polymer D at 5 mg/mL: Zero slope at low q indicates spherical nanoparticles. The red line is model fit for fuzzy sphere. (E) The sharp peak in the Kratky plot for polymer D at 5, 2, and 1 mg/mL validates compact self‐assembly. (F) A Guinier plot (linear region of ln(*I*) vs. q^2^ at q∼0) to calculate *R*_g_ of the self‐assembled nanoparticles using the formula *R*_g_ = √‐3slope, results in a *R*_g_ of 16.8 nm for polymer D at 37°C. (G) Cryo‐TEM of polymer D at 37°C, blue arrows point to nanoparticles; scale bar = 200 nm. (H) Effect of concentration on *T*_t_ for diblock POEGMAs: the concentration dependence on *T*_t_ of polymers that do not exhibit self‐assembly is much more prominent than the ones that self‐assemble. (I) Boundary conditions for self‐assembly of POEGMA diblock copolymers. The relative length of each block needs to be at least 25% of the total chain length to impart enough amphiphilicity to the diblock for its self‐assembly in aqueous media.

Each POEGMA diblock copolymer in the library was characterized for evidence of self‐assembly. For simplicity, we show the physical characterization data for only one polymer, polymer D, as a representative of this diblock library in Figure 2, but the physical parameters for other polymers are summarized in Table 1, and the characterization data are shown in Supporting Information (Figures S1–S5).

**TABLE 1:** Library of EG3‐EG2 diblock POEGMA, where each block is a homopolymer.

Label	^a^ID	^a^M_n_	*T*_t_ (°C)	∼CMT (°C)	^b^d_h_ ± SD (nm)	^c^ *R*_g_ ± SE (nm)	^c^Shape
A	EG3_22_‐EG2_119_	27 540	30	n/a	6 ± 2	n/m	n/a
B	EG3_73_‐EG2_70_	30 107	43	35	30 ± 9	10.7 ± 1.09	spherical
C	EG3_73_‐EG2_225_	59 280	44	28	92.5 ± 25	could not be deduced	cylindrical
D	EG3_167_‐EG2_85_	54 810	42.5	31	40 ± 10	16.8 ± 1.45	spherical
E	EG3_134_‐EG2_34_	37 630	45	n/a	6.5 ± 2.5	4 ± 1.33	n/a

^a^ Determined by SEC‐MALS;

^b^ DLS;

^c^ SAXS at temperature between CMT and *T*_t_; n/m: not measured. The *T*_t_ is reported at 100 µM and CMT is reported at 50 µM.

First, the LCST phase behavior of all the diblock copolymers was studied by measuring their absorbance at 600 nm as a function of solution temperature at various concentrations in PBS, and their *T*_t_ were determined by the temperature at the inflection point in the absorbance as a function of heating. To test the reversibility of the LCST phase behavior of the diblock POEGMA, the polymers were also cooled at the same rate while measuring their absorbance. As expected, the absorbance of all polymers returned to their baseline after cooling, indicating completely reversible phase transition behavior but with slight differences in their thermal hysteresis. Based on the absorbance as a function of temperature, we determined a *T*_t_ of 42.5°C for polymer D at 100 µM, corresponding to the temperature at the inflection in its absorbance (Figure 2A).

Second, all the diblock polymers were characterized by dynamic light scattering (DLS) at various concentrations below their *T*_t_ to study if they exhibit self‐assembly. For diblock polymer D in the concentration range of 10–200 µM, the hydrodynamic diameter (d_h_ ± SD) is 7 ± 2 nm at 25°C, corresponding to the unimer of POEGMA, but increases to 40 ± 10 nm upon heating to 30°C, indicating temperature‐dependent self‐assembly of the polymer into nanoparticles. This temperature also corresponds to the small hump in the absorbance of the polymer at 28°C–31°C and is the critical micellization temperature (CMT) of polymer D. The size for polymer D at 25°C and at body temperature shows a single peak with normal distribution at both a low (12.5 µM) and at a high (200 µM) concentration (Figure 2B). The 40 ± 10 nm size of the self‐assembled nanoparticles is stable over a wide range of temperatures for polymer D above its CMT until the nanoparticle to coacervate phase transition occurs at the *T*_t_ of the polymer at 42.5°C (Figure 2C).

To further characterize the self‐assembly behavior of polymer D, small‐angle X‐ray scattering (SAXS) measurements at 37°C were performed. The 1D SAXS curve (Figure 2D) displays a zero slope at small scattering vectors (q), indicative of spherical nanoparticles, which is also validated by the sharp peak in the Kratky plot (Figure 2E), indicating a compact assembly [21]. The radius of gyration (*R*_g_) was calculated from the Guinier plot (Figure 2F) and was 16.8 ± 1.45 nm. In good agreement with DLS, the size and shape of the nanoparticles did not change with varying polymer concentration in the range of 1–5 mg/mL, corresponding to ~20–100 µM. The 1D SAXS curve could be fitted to the fuzzy sphere model with an overall particle radius of 20 nm (Figure 2D) validating the expected micellar structure [22]. From the model, the radius of the compact particle is 17.1 ± 0.15 nm surrounded by fuzziness of 3 ± 0.17 nm.

To visualize the diblock POEGMA nanoparticles in their hydrated state, we imaged them with cryo‐transmission electron microscopy (cryo‐TEM) at 37°C—a biologically relevant temperature and a temperature between its CMT and *T*_t_. The electron contrast of these polymeric nanoparticles is low compared to the solvent due to their highly hydrated corona, allowing us to visualize only the core structure of the nanoparticles. Figure 2G shows a cryo‐TEM image of polymer D at 37°C, where the blue arrows point to a few of the nanoparticles in the micrograph. Image J analysis of 50 particles from 5 separate micrographs resulted in a diameter of about 18 ± 3 nm for polymer D.

Overall, these results show that even the difference of a single EG side chain in each block of an EG3‐EG2 diblock copolymers imparts sufficient amphiphilicity to a subset of these diblock copolymers to drive their self‐assembly into nanoparticles. Polymer B, C and D, show strong self‐assembly in response to temperature with a similar *T*_t_ ~ 43°C ± 1°C, irrespective of the overall hydrophobic composition, whereas polymer A and E remain unimers at all temperatures with distinct *T*_t_ that corresponds to the overall hydrophobic composition of the polymer (Table 1). We propose that the *T*_t_ of the self‐assembling block‐copolymers is dependent only on the composition of the corona forming block and not on the overall hydrophobicity. On the other hand, the block copolymers that do not self‐assemble resemble random copolymers of EG3 and EG2, where the *T*_t_ is inversely proportional to the overall hydrophobicity of the polymer. Additionally, the *T*_t_ of several EG3‐EG2 diblock copolymers —notably B and D— have a very low to no dependence on the polymer concentration as compared to polymers A and E. We suggest that nanoparticle assembly below the *T*_t_ explains the concentration independence of B and D (Figure 2H). This is consistent with similar behavior seen in diblocks of elastin‐like polypeptides that also show LCST phase behavior [23, 24], where the steep dependence of the *T*_t_ as a function of concentration is a signature of a unimer to coacervate phase transition, whereas polymers that show a low dependence of *T*_t_ on concentration is reflective of self‐assembly at a temperature below their *T*_t_ as the polymer exhibits 2‐step transition from a soluble unimer to nanoparticle, and then from nanoparticle to the coacervate phase.

To determine the boundary conditions for self‐assembly of the EG3‐EG2 diblock copolymers, we plotted the relative EG2 block lengths in terms of their DP against the relative EG3 block length (Figure 2I). The area outside the green box represents compositions that are unimers at all temperatures below *T*_t_ whereas the area within the box represents diblock compositions that self‐assemble into nanoparticles in response to elevated temperature below their *T*_t_. We determined that the relative DP of an individual block in a simple EG3‐EG2 diblock copolymer needs to be at least 25% of the total DP to impart enough amphiphilicity to the copolymer to drive its self‐assembly into nanoparticles.

In summary, the data for the diblock library in Table 1, where each block is an EG2 or EG3 homopolymer, shows that self‐assembly of these EG3‐EG2 diblock POEGMA can be achieved by varying the relative block lengths to more than 25% of the total DP, but that all these polymers require a temperature higher than room temperature (RT) for self‐assembly to occur.

### 2.2 Increasing the Hydrophobicity of the Hydrophobic Block to Access Stable Nanoparticles at Room Temperature for Drug Delivery

**FIGURE 3:**
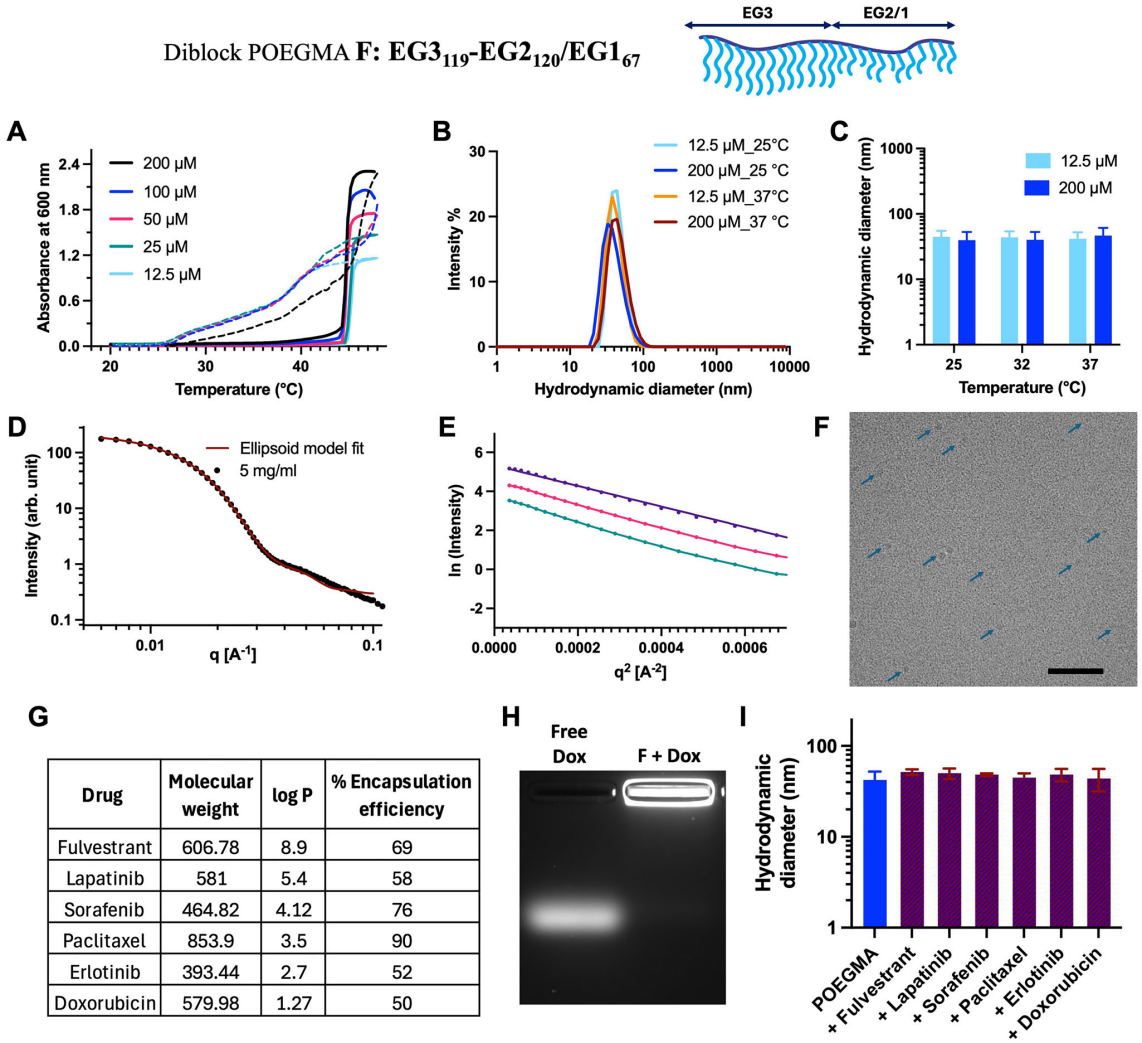
Optimal POEGMA diblock composition for physical drug encapsulation. Polymer F—diblock copolymer with a hydrophilic EG3 homopolymer block and a hydrophobic block that is a random copolymer of EG1 and EG2 monomers—provides robust nanoparticles with a desirable micellization temperature of below room temperature. (A) Absorbance at 600 nm of polymer F as a function of solution temperature at various polymer concentrations in PBS. Heating curves are represented by solid lines, and cooling curves are represented by dashed lines. A sharp increase in absorbance at 44.5°C indicates the *T*_t_ for polymer F for the transition from nanoparticle to coacervate phase. (B) The size distribution by DLS at room temperature and body temperature for polymer F at two different concentrations. (C) Hydrodynamic diameter (d_h_) of polymer F for a wide range of relevant temperatures. (D) 1D SAXS plot at 25°C for polymer F at 5 mg/mL. The model fit (red line) indicate ellipsoid shape. (E) A Guinier plot to calculate *R*_g_ of the self‐assembled Nanoparticles, resulting in a *R*_g_ = 12.6 nm for polymer F at 25°C. (F) cryo‐TEM of polymer F at RT. Blue arrows point to nanoparticles; scale bar is 100 nm. (G) Percent encapsulation efficiency of various small molecule chemotherapeutics with a wide range of log P values in polymer F. (H) Agarose gel showing successful encapsulation of Dox: free Dox (left) and Dox encapsulated in polymer F (right). (I) Hydrodynamic diameter measured by DLS shows that drug encapsulation does not affect the size and stability of the POEGMA nanoparticles. The DLS measurements were recorded at least 3‐times, and the data is presented as mean ± SEM.

To achieve self‐assembly of POEGMA diblock copolymers at room temperature and hence access nanoparticles that are stable from room temperature to body temperature, a temperature range that is useful for biomedical applications, we next synthesized a more complex library of diblock POEGMA with a greater range of amphiphilicity by doping the more hydrophobic EG1MA monomer into the hydrophobic block (Table 2). Polymers F, G, H, and I belong to this class, where the hydrophobic, core forming block is a random copolymer of EG1MA and EG2MA. For simplicity, we show the physical characterization data for only one representative polymer from this diblock library—polymer F—in Figure 3, but the data for the other polymers are summarized in Table 2 and are shown in the Supporting Information (Figures S6–S9).

**TABLE 2:** Library of an EG3 ‐EG2/EG1 diblock copolymer with a hydrophilic block that is an EG3 homopolymer and a hydrophobic block that is a random copolymer of EG1 and EG2 monomers.

Label	^a^ID	^a^M_n_	^a^M_w_	^a^ *D_m_*	*T*_t_ (°C)	∼CMT (°C)	^b^d_h_ ± SD (nm)	^c^ *R*_g_ ± SE (nm)	^c^Shape
F	EG3_119_‐EG2_120_/EG1_67_	60 030	77 030	1.28	44.5	<rt	44 ± 10.6	12.6 ± 1.4	ellipsoid
G	EG3_154_‐EG2_95_/EG1_82_	65 590	91 250	1.39	43.5	<rt	56 ± 15	n/m	n/m
H	EG3_63_‐EG2_54_/EG1_18_	27 290	33 670	1.23	44.5	<rt	47 ± 21	n/m	n/m
I	EG3_68_‐EG2_42_/EG1_21_	26 795	29 810	1.11	45	<rt	23.3 ± 8	7 ± 1.3	Porous and semiflexible

^a^ Determined by SEC‐MALS;

^b^ DLS;

^c^ SAXS; n/m: not measured. The *T*_t_ is reported at 100 µM and CMT is reported at 50 µM.

First, the LCST phase behavior of polymer F at various concentrations is shown in Figure 3A, and the polymer has a *T*_t_ of 44.5°C at a concentration of 100 µM. Polymer F shows completely reversible phase transition behavior, but has more hysteresis compared to polymer D that appears to be related to its greater overall hydrophobicity, compared to polymer D. Interestingly, despite having variable amounts of the hydrophobic EG1 monomer, the *T*_t_ for all self‐assembling polymers in this set are ~43°C–44°C, irrespective of the EG1 doping levels (Table 2). This can be attributed to the fact that any differences in the hydrophobic core are masked by the similar EG3 corona in all these polymers, whose desolvation with increasing temperature drives the self‐assembled polymer nanoparticles to coacervate, an effect that has also been seen with genetically encoded diblock copolymers of elastin‐like polypeptides that also exhibit LCST phase behavior [23, 24]. This observation further supports the notion that the phase transition temperature is predominantly dictated by the composition and hydration dynamics of the hydrophilic block rather than the hydrophobic core. While the EG1MA incorporation modulates core hydrophobicity and influences micelle stability and size distribution, its contribution to *T*_t_ appears secondary to that of the EG3 corona. The cooperative dehydration of the EG3 side chains upon heating triggers the onset of coacervation, effectively overriding subtle differences in the core composition. Together, these results highlight that tuning the hydrophilic block architecture provides a more direct handle for precise control over the LCST behavior of POEGMA diblock copolymers. Second, DLS of these polymers reveals that their CMT is below RT, and that they show stable self‐assembly at a wide range of concentrations ranging from 12.5 to 200 µM and over a wide range of temperatures up to their *T*_t_ of ~44°C (Table 2). The size by DLS for polymer F at RT and body temperature shows a normal distribution with a single peak at a d_h_ of 44 ± 10 nm for a wide range of polymer concentrations in PBS (Figure 3B,C).

To further study the self‐assembly behavior of polymer F at RT, SAXS was carried out at concentrations of 1, 2, and 5 mg/mL in PBS. The near‐zero slope of the 1D SAXS curve at small q suggested nearly spherical nanoparticles. The scattering data could be fitted to an ellipsoid model, with a polar radius of 10.6 nm and an equatorial radius of 17.3 nm. These parameters indicate that the nano‐assembly is not perfectly globular but exhibits a modestly elongated morphology (Figure 3D). The sharp peak in the Kratky plot (Figure 6B) indicated a compact assembly. The *R*_g_ of self‐assembled nanoparticles of polymer F was determined using Guinier approximation and was found to be 12.6 ± 1.37 nm (Figure 3E). Polymer F was further visualized by cryo‐TEM at RT (Figure 3F). As previously, we suspect that only the tightly packed core of these nanoparticles is visible, rather than the whole nanoparticle due to its highly hydrated EG3 corona. The blue arrows point to a few representative nanoparticles on the micrograph in Figure 3F, and Image J analysis of 50 particles from 5 separate micrographs resulted in a diameter of 12.2 ± 4 nm for polymer F.

Overall, the data for the four complex diblocks—polymers F, G, H, I— show that the diblock POEGMA composition can be easily manipulated to create thermo‐responsive block polymers that self‐assemble into nanoparticles that are stable from below room temperature to above body temperature. Additionally, to demonstrate that the hydrophobic end‐group from the chain transfer agent (cta) on these RAFT synthesized polymers has no impact on the self‐assembly, we cleaved‐off the CTA group from polymer F by treatment with excess free radicals, and the reaction was monitored by discoloration of the reaction mixture [25]. Cleavage of the CTA end group had no impact on the CMT, size or shape of the nanoparticles that are formed by self‐assembly of the polymer nor the nanoparticle to coacervate phase transition behavior (Figure S10).

Next, to showcase the utility of these nanoparticles, we physically encapsulated various small cancer drugs with a wide range of hydrophobicity —as seen by their log P values—in nanoparticles of polymer F by simple incubation of the polymer with a 10‐fold molar excess of the drug. The free drug was separated from the encapsulated drug by ultrafiltration, and the encapsulation efficiency of the drugs was monitored by measuring the absorbance of free drug versus encapsulated drug by HPLC. We find that various small hydrophobic drugs with logP range ∼ 1–9 can be encapsulated in nanoparticles of block copolymer F by simple mixing in PBS with an encapsulation efficiency of 50%–80% (Figure 3G). The encapsulation efficiency of a drug can be further improved by optimizing the encapsulation parameters such as solvent, pH, drug to polymer equivalent ratio, and incubation time, which we only explored for a single drug —the potent anti‐cancer drug Paclitaxel—in this preliminary study. We observed encapsulation efficiency of greater than 90% for Paclitaxel (PTX) by both thin film hydration and buffer exchange methods at a drug to polymer ratio of 10:1. The hydrodynamic size of the POEGMA nanoparticles before and after drug encapsulation was found to be unaffected, as observed by DLS (Figure 3I and Figure S13). Out of the six drugs tested for encapsulation, we decided to proceed with Doxorubicin (Dox) to in vitro and in vivo studies, as the intrinsic fluorescence of Dox can be easily tracked.

The encapsulated Dox in polymer F was also confirmed by agarose gel electrophoresis by visualizing its fluorescence (Figure 3H). The positively charged free Dox migrates toward the negative electrode in an agarose gel, whereas its charge is masked when encapsulated in the neutral polymeric nanoparticles, and hence the encapsulated Dox stays in the well. Additionally, to assess the colloidal stability of the POEGMA assemblies under physiologically relevant conditions, we performed serum‐stability studies by incubating both empty and Dox‐loaded nanoparticles in medium containing 20% serum at 37°C. Before nanoparticle exposure, the scattering profile of serum alone was recorded to quantify background contributions and ensure accurate interpretation of DLS measurements. Across 168 h of incubation, the size distribution of the POEGMA nanoparticles did not change, demonstrating that the assemblies remain colloidally stable in the presence of serum proteins (Figure S21). These findings indicate that the POEGMA nanoparticles resist aggregation, fusion, or premature disassembly under conditions that mimic systemic circulation, supporting their suitability for in vivo applications.

To evaluate the release behavior of doxorubicin (Dox) from POEGMA nanoparticles, we conducted drug release assays at 37°C in four buffer conditions representative of biological environments: (1) physiological pH (7.4); (2) tumor microenvironment pH (6.5–7.0); (3) late endosomal/lysosomal pH (~5.0). Dox‐loaded POEGMA nanoparticles and free Dox controls were placed inside dialysis cassettes (MWCO 3.5 kDa) and dialyzed against respective buffers for a week. Aliquots were collected at 0, 1, 2, 4, 8, 24, 48, 72, 120, 144, and 168 h and quantified by fluorescence using a Dox calibration curve. Consistent with its small molecular size, free Dox rapidly diffused out of the dialysis cassettes within the first 10 h, with minimal pH dependence (Figure S14A). In contrast, Dox encapsulated in POEGMA nanoparticles was strongly retained, with a substantial fraction remaining inside the cassette even after 168 h. Release was modestly accelerated at lower pH, consistent with increased solubility of free Dox at lower pH (Figure 14B). These results support slow‐release kinetics, and the strong drug retention in the nanoparticle over one week suggests that drug release is driven primarily by diffusion from a stable hydrophobic core, with mild pH sensitivity.

To evaluate the biological safety of POEGMA, we assessed the in vitro cytotoxicity of a representative polymer—POEGMA ‘F’—using two murine colorectal adenocarcinoma cell lines— MC38 and CT26. Cells were seeded in 96‐well plates and treated with increasing concentrations of POEGMA (0–250 µM) for 72 h. Cell viability was quantified using a metabolic MTS assay, and all measurements were normalized to untreated controls. Across the entire concentration range, POEGMA ‘F’ exhibited no measurable cytotoxicity in either cell line (Figure S15), indicating that the polymer is well‐tolerated and does not adversely affect cellular metabolic activity or overall health. These data further support the biocompatibility of POEGMA and its suitability for biomedical applications.

**FIGURE 4:**
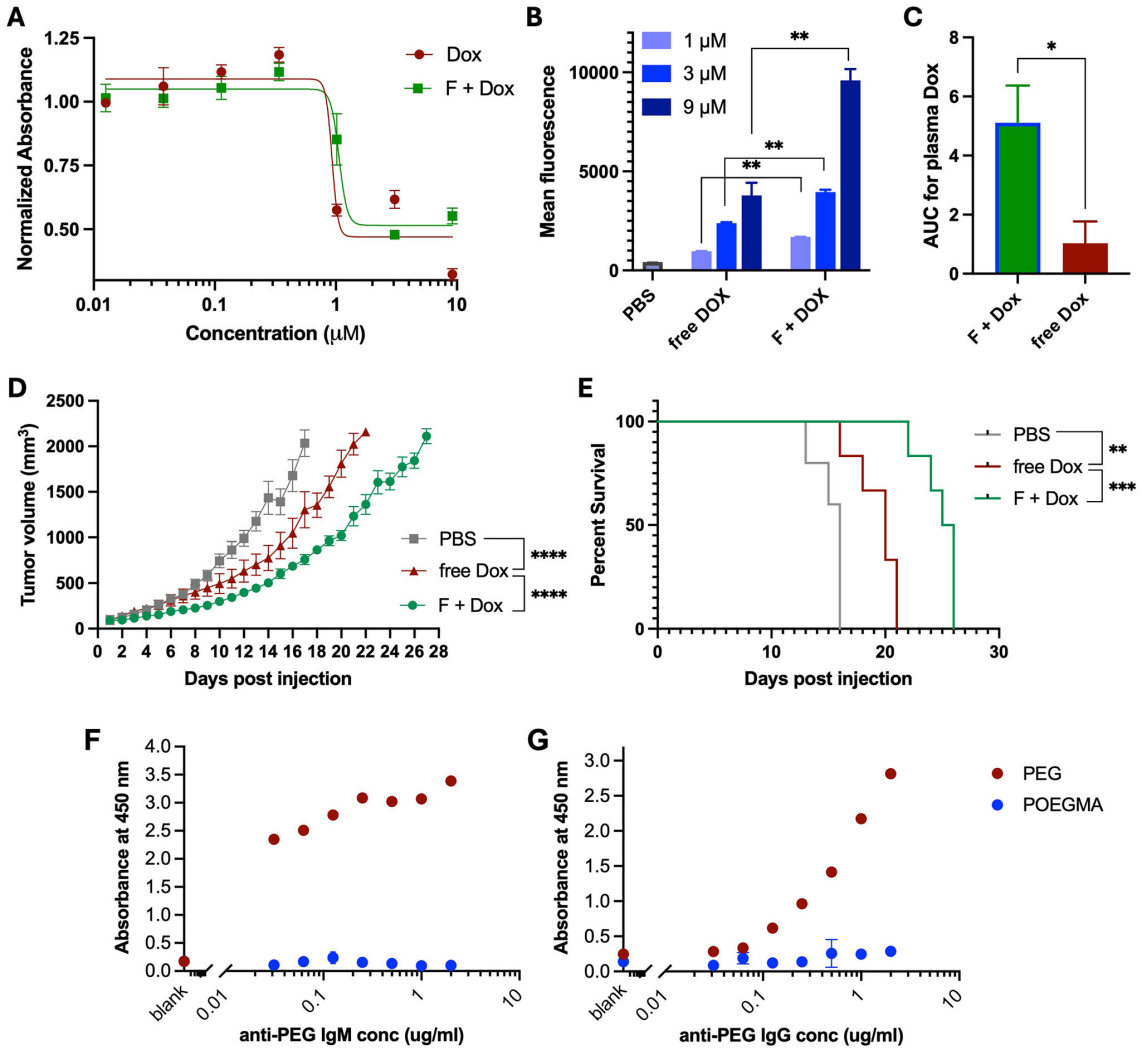
Utility of diblock POEGMA in Dox encapsulation and its efficacy. (A) In vitro potency of Dox in a C26 colon carcinoma cell line after 72 h incubation with the free drug or drug encapsulated in polymer F (n = 3). (B) Uptake of encapsulated Dox in polymer F and free Dox at Dox equivalent concentrations of 1, 3 and 9 µm by the C26 tumor cell line in vitro after 24 h of incubation, as quantified by flow cytometry (n = 3). (C) Total drug exposure—AUC—of Dox in plasma of mice (n = 3) after intravenous administration of free Dox or encapsulated Dox in polymer F at an equivalent Dox dose of 4 mg/kg. (D,E) Anti‐tumor efficacy of the Dox encapsulated in POEGMA F as compared to the free Dox in terms of (D) change in primary tumor volume and (E) Kaplan–Meier survival of mice post‐treatment at a Dox equivalent dose of 10 mg/kg in mice bearing syngeneic C26 (n = 6). Significance of the data was analyzed by two‐way ANOVA (Tukey's test) for tumor regression and log‐rank (Mantel‐Cox) test for survival. Data is presented as mean ± SEM, ^**^ *p* < 0.01, ^***^ *p* < 0.001, and ^****^ *p* < 0.001. (F,G) POEGMA nanoparticles showing negligible binding to preexisting anti‐PEG IgM (F) and IgG (G) antibodies as compared to PEG as determined in an indirect ELISA assay (n = 3), confirming preserved stealth behavior.

Next, the cytotoxicity of free Dox and encapsulated Dox in polymer F was also evaluated in the C26 and MC38 cell lines by the in vitro MTS assay (Figure 4A and Figure S16, respectively). Free Dox is more cytotoxic at early time points, but the IC_50_ of encapsulated Dox catches up by 72 h after incubation (Figure S16). We further studied the uptake of nanoparticles in vitro by confocal fluorescence microscopy by monitoring the red fluorescence of Dox and the blue fluorescence of cell nuclei stained with Hoechst blue dye in both the C26 and MC38 cell lines. Interestingly, more red fluorescence was observed in the cytoplasm for encapsulated Dox as compared to free Dox after incubation for 24 h (Figures S18 and S19). To validate and quantify this observation, we also studied uptake of Dox by C26 cells by flow cytometry at a Dox concentration of 1, 3 and 9 µM after 24 h, which revealed that the cellular uptake of encapsulated Dox in polymer F is 1.5–3 times greater than that of free Dox (Figure 4B). Based on these observations, we suspect that the uptake of nanoparticles by cells is not the rate‐limiting step in the decreased cytotoxicity of the encapsulated Dox as compared to free Dox. Instead, we believe that the lower cytotoxicity is caused by the fact that these nanoparticles are taken up by macropinocytosis and end up in the endosomes, from where the drug has to escape the nanoparticle and diffuse out of endosomes into the cytoplasm and then traffic to the primary site of activity of the drug—the nucleus [26]. This is an inherently inefficient process that is only partly compensated for by the higher overall level of uptake of the drug that is encapsulated in the polymer nanoparticles relative to the free drug.

To further investigate the in vivo properties of Dox‐loaded in POEGMA nanoparticles, we measured their pharmacokinetics (PK) and carried out an in vivo efficacy study in mice. For the PK study, Dox fluorescence was monitored in plasma collected at various time points after intravenous (*i.v*.) injection of free Dox or Dox encapsulated in polymer F (Figure S22). The half‐life (*t*_1/2_) of drug's elimination was calculated by fitting a one‐phase decay model to the PK curve. The *t*_1/2_ for free Dox vs encapsulated Dox was found to be 1.5 vs 8.5 min, respectively. Overall, the total drug exposure in plasma calculated by area under the PK curve (AUC) for encapsulated Dox was five‐fold higher as compared to the free Dox (Figure 4C). Interestingly, the plasma Dox concentration for encapsulated Dox was five‐fold lower than that of free Dox 45 s after injection, suggestive of higher uptake and hence significantly altered biodistribution of Dox (Figure S22).

To evaluate the anti‐cancer efficacy of encapsulated Dox in POEGMA nanoparticles relative to free Dox, we used the Dox‐sensitive and rapidly growing syngeneic C26 tumor model in mice. A single dose of free Dox or Dox encapsulated in polymer F was injected via tail vein after the subcutaneous (*s.c*.) tumors reached 75–100 mm^3^ in size. Both treatments were injected at the previously reported maximum tolerated dose (MTD) of 10 mg/kg Dox equivalent [27]. The tumor size and body weight were monitored daily until the tumor volume exceeded 2000 mm^3^, at which point the mice were euthanized. The encapsulated Dox showed superior efficacy as compared to the free Dox in terms of tumor regression (*p* < 0.0001, two‐way ANOVA and Tukey's test) (Figure 4D) and survival (*p* < 0.001, log‐rank (Mantel‐Cox) test) (Figure 4E) at an equivalent dose.

We also investigated the anti‐PEG antigenicity of our diblock POEGMA nanoparticles in an indirect ELISA assay format. Briefly, the antigen—PEG (positive control) or POEGMA—coated plate was incubated with commercially available anti‐PEG IgM or IgG antibodies at various concentrations, and the binding events were monitored by recording absorbance at 450 nm. While PEG binds to anti‐PEG IgG and IgM very strongly in a dose‐dependent manner, negligible binding of diblock POEGMA to anti‐PEG IgG or IgM was observed even at very high concentrations (Figure 4F,G).

## 3 Conclusions

In this work, we exploited the LCST phase behavior of POEGMA to design and synthesize polymeric nanoparticles composed solely of POEGMA. Given that the *T*_t_ of POEGMA can be tuned by varying the EG side‐chain length, we designed diblock POEGMA copolymers with differing EG lengths to achieve sufficient amphiphilicity for self‐assembly in aqueous media. Based on our previous findings, we restricted the EG repeat units in the monomers to three or fewer to ensure stealth properties while avoiding binding to anti‐PEG antibodies. We synthesized a library of simple AB‐type POEGMA block copolymers using EG3MA and EG2MA and showed that a single EG unit difference is sufficient to impart amphiphilicity for self‐assembly. Varying the relative block lengths revealed that each block must contribute at least 25% of the total polymer length for thermoresponsive micellization. Partial phase diagrams showed that diblocks unable to self‐assemble exhibit concentration‐dependent *T*_t_ as they transition from unimer‐to‐coacervate, whereas self‐assembling diblocks display concentration‐independent *T*_t_ as they transition from micelle‐to‐coacervate phase. We further showed that EG3MA/EG2MA diblocks remain unimeric at room temperature but can self‐assemble at slightly elevated temperatures. To expand the temperature range of stability, we increased the hydrophobicity of the hydrophobic block by incorporating EG1MA through random copolymerization, resulting in nanoparticles that are stable at both room temperature and body temperature. We demonstrate a case for the utility of these self‐POEGMA nanoparticles by encapsulating a variety of clinically relevant small hydrophobic drugs with high loading efficiency. We further demonstrate that the POEGMA nanoparticle encapsulating doxorubicin is pharmacologically active in vitro and shows superior efficacy in vivo due to its improved pharmacokinetics as compared to free doxorubicin. Lastly, we confirmed that these POEGMA diblock nanoparticles do not bind to anti‐PEG antibodies, highlighting their potential as a stealth alternative to PEG for drug delivery applications.

## 4 Outlook

This study establishes a new class of self‐assembling nanoparticles composed entirely of POEGMA, offering a modular and stealth‐compatible platform for drug delivery. By leveraging the tunable thermoresponsive behavior of POEGMA and its side‐chain‐dependent amphiphilicity, we have demonstrated precise control over nanoparticle assembly, temperature sensitivity, and drug encapsulation capabilities without relying on any other hydrophobic comonomers to drive self‐assembly. Additionally, the ability to avoid anti‐PEG antibody recognition while retaining desirable pharmacokinetics marks a significant advance in the development of next‐generation stealth nanocarriers. The synthetic simplicity and versatility of POEGMA chemistry also open possibilities for large‐scale production and clinical translation. Future work will focus on expanding the platform to encapsulate biologics, exploring alternative administration routes, and conducting detailed immunological studies in clinically relevant models. Overall, this work lays the foundation for the development of safe, efficient, and customizable POEGMA‐based nanocarriers that can address the limitations of traditional PEGylated nanocarriers.

## 5 Methods

### 5.1 Materials

All reagents for the synthesis of the block coPOEGMA were purchased from Sigma Aldrich. The monomers—triethylene glycol methyl ether methacrylate (EG3MA, CAS No. 24493‐59‐2), diethylene glycol methyl ether methacrylate (EG2MA, CAS No. 45103‐58‐0), ethylene glycol methyl ether methacrylate (EG1MA, CAS No. 6976‐93‐8)—were passed through a column of activated basic aluminum oxide (CAS No. 1344‐28‐1, Sigma # 199443) to remove inhibitors before polymerization reactions. 4‐Cyano‐4‐(thiobenzoylthio) pentanoic acid (CAS 201611‐92‐9, Sigma # 722995,) and 2,2’‐Azobis (2‐methyl propionitrile) (AIBN) (CAS 78‐67‐1, Sigma # 441090,) and solvents were used as received. Sorafenib, Erlotinib, Lapatinib, and Fulvestrant were purchased from MedChemExpress (MCE). Paclitaxel was purchased from Ark Pharm (Arlington Heights, IL), and Doxorubicin HCl was purchased from Biosynth (Gardner, MA).

### 5.2 Synthesis of Diblock POEGMA Copolymers

POEGMA diblock copolymers were synthesized in a two‐step sequence of reversible addition‐fragmentation chain‐transfer (RAFT) polymerization employing 4‐Cyano‐4‐(thiobenzoylthio) pentanoic acid as the chain transfer agent (CTA) and AIBN as the free radical initiator. The first block (EG3 POEGMA) was polymerized by mixing the EG3MA monomer, CTA, AIBN in 4x:4:1 molar equivalent ratio, respectively, in toluene as a solvent, where x is 1.2 times the desired degree of polymerization (DP) of the monomer. The reaction mixture was prepared in a round bottom (RB) flask on an ice bath and purged with nitrogen gas for 40 min to create an inert environment before initiating the polymerization at 70°C for a specified time with continuous nitrogen purging. The reaction time ranged from 1–5 h depending on the target molecular weight and the initial monomer concentration. The reaction was quenched by submersion of the RB flask in liquid nitrogen, and the resulting EG3 polymer was purified with three rounds of hexane precipitation. The purified polymer was freeze‐dried and stored at −20°C until further use. To complete the synthesis of the diblock copolymer, the second block (EG2 POEGMA) was polymerized by utilizing the EG3‐POEGMA block as a macromolecular CTA. To do so, the first polymer block was resuspended in toluene, and the EG2MA monomer and AIBN were added to the RB flask on an ice bath (Figure 1B). The reaction conditions and purification procedure were identical to those used for the EG3 polymerization. For the second set of the diblock polymers shown in Table 2, the individual blocks were synthesized and purified similarly except that a mixture of the EG2MA and EG1MA monomers was randomly copolymerized in the hydrophobic block.

### 5.3 CTA End Group Removal

The active part— thiobenzoylthio —of the CTA present as an end group on RAFT synthesized polymers was cleaved off from the polymer chain by treatment with a 20‐fold molar excess of AIBN at 80°C for 3 h in an inert environment, resulting in a colorless solution. The CTA‐free polymer was purified by three rounds of hexane precipitation.

### 5.4 Chemical Characterization of POEGMA

All polymerization reactions were monitored with proton nuclear magnetic resonance (^1^H NMR) spectroscopy on a 500 MHz NMR spectrometer (Varian) to determine the percent monomer to polymer conversion and determine the molecular mass. After quenching the polymerization reaction, a small amount of the mixture was dissolved in deuterated chloroform (CDCl_3_) containing 1% (v/v) tetramethylsilane (TMS) as an internal reference to record the ^1^H NMR spectrum. The NMR data were analyzed using MNOVA software. All purified polymers were additionally characterized for number average and weight average molecular weights—M_n_ and M_w_ respectively—and molar mass distribution (*D_m_*) by size exclusion chromatography in line with multi angle light scattering (SEC‐MALS) using an Agilent 1260 Infinity HPLC equipped with a DAWN HELEOS II MALS detector and an Optilab T‐rEX refractive index detector (Wyatt Technology). The polymers were resuspended in HPLC grade tetrahydrofuran (THF) at a concentration of 2–5 mg/mL and filtered through 0.2 µm syringe filters (Whatman). Each sample (50 µL) was injected into dual Agilent gel columns (7.5 × 300 mm, 1 79911GP‐503 (103 Å) and a 1 79911GP‐504 (104 Å) and run at room temperature using THF (containing 100 ppm butylated hydroxytoluene (BHT) as a stabilizer) as the mobile phase at a flow rate of 1 mL/min. The light scattering data were analyzed for M_n_ and M_w_ using ASTRA software (Wyatt) by inputting the refracting index increment (dn/dc) = 0.07 for POEGMA in THF. The dn/dc value for POEGMA was calculated using the refractive index detector with an online method for a known concentration of the polymer sample.

### 5.5 LCST Phase Behavior and Hydrodynamic Size

The polymers were resuspended in 1x PBS at 200 µM concentration at 4°C, and a serial dilution in PBS was performed resulting in 200, 100, 50, 25, 12.5, 6.25 µM as the concentrations to be tested. The optical density (OD) of all polymers was monitored at 600 nm as a function of temperature on a temperature‐controlled UV–vis spectrophotometer (Cary 300 Bio, Varian instruments). Starting at 20°C, the temperature of the samples in the cuvette was increased at a rate of 0.3°C/min until ~ 48°C and was then cooled back to 20°C at a rate of 0.3°C/min. The absorbance was recorded at each 0.6°C intervals. A sharp increase in the OD with temperature is indicative of the phase transition, and the temperature at the inflection point of the optical density is defined as the *T*_t_. The OD vs temperature was plotted, and the temperature at the inflection point that defines the *T*_t_ was determined by finding the maximum of the first derivative of the OD versus temperature using GraphPad Prism software. The size distribution of the diblock POEGMA samples was measured using a Malvern NanoZS Zetasizer equipped with a 173° backward scatter detector. The DLS measurements were performed in triplicate at various temperatures for various concentrations in a low‐volume quartz cuvette (Malvern #ZEN2112). Data were analyzed by a regularization fit for Raleigh spheres using Malvern sizing software. A combination of the UV–vis spectrophotometry and the DLS data was used to determine the critical micellization temperature (CMT).

### 5.6 Small Angle X‐Ray Scattering

SAXS data were collected at the 16ID‐LiX Beamline of the National Synchrotron Light Source II (NSLS‐II), Brookhaven National Laboratory, Upton, NY. The X‐ray wavelength was set to 0.8189 Å, and both small‐ and wide‐angle X‐ray scattering (SAXS and WAXS) setups were utilized simultaneously to cover scattering *q* ranges of 0.006 ≤ *q* ≤ 3.19 Å^−1^, where *q* = (4π/λ)sinθ, with 2θ representing the scattering angle and λ indicating the X‐ray wavelength. For room temperature measurements, the sample solution and matching buffer were measured in a capillary flow cell at room temperature, and data frames (10–16) were recorded with a 0.5 s exposure time per frame while the sample flowed in a single direction. For temperature‐dependent measurements at 25°C and 37°C, custom‐designed flat fixed cells with mica windows and temperature control capabilities were employed. Before each solution scattering measurement, both the empty cells and the matching buffer were measured for proper background subtraction. To minimize radiation damage, twenty repeated measurements were performed at different positions on the sample, with each frame taken at a 0.5 s exposure time. The 2D scattering images collected by the SAXS and WAXS detectors were converted into 1D scattering profiles and merged. Transmission corrections and background subtraction were applied to minimize the water peak at ~2.0 Å^−1^. The final scattering profiles were obtained by averaging the repeated measurements after eliminating outliers and subtracting contributions from the matching buffer. Data processing was performed using LiXTools (https://github.com/NSLS‐II‐LIX/lixtools) and the Python package py4xs (https://github.com/NSLS‐II‐LIX/py4xs) in NSLS‐II Jupyter Notebook environment [28]. Final scattering profiles were also manually validated using the Irena package for analysis of small angle scattering data [29]. Model fitting was performed by fitting the scattering curve to an appropriate model by SasView program [30]. To calculate the radius of gyration (*R*_g_), we employed the Guinier approximation that at low q, the scattering profile can be approximated as I(q) ≈ I(0) e(‐q^2^ *R*_g_^2^/3), where I(0) is the intensity at zero scattering angle. A linear fit to a plot of ln(I) vs. q^2^ (Guinier plot) in the linear fitting region gives the formula *R*_g_  =  √ − 3a, where ‘a’ is the slope of the Guinier fit.

### 5.7 Cryo‐TEM

POEGMA samples were resuspended in 1x PBS at concentrations of 1, 2, and 5 mg/mL and imaged by TEM at cryogenic temperature for nanoparticle structure analysis below the *T*_t_ of the polymer. Vitrified specimens were prepared on a copper grid coated with a perforated lacey carbon 300 mesh (Ted Pella Inc.). A drop (3 µL) from the solution was applied to the grid in a controlled environment (with temperature set to either room temperature or 37°C) and blotted with a filter paper to form a thin liquid film of solution. The blotted samples were immediately plunged into liquid ethane at its freezing point (−183°C). The procedure was performed automatically in the Plunger (Leica EM GP). The vitrified specimens were then transferred into liquid nitrogen for storage. The samples were studied using a FEI Talos F200C TEM, 200 kV maintained at liquid nitrogen temperature; and images were recorded by FEI Ceta16M camera (4k × 4k CMOS sensor) at low dose conditions, to minimize electron beam radiation damage. The particle size was analyzed manually by measuring the diameters of nanoparticles in ImageJ. Briefly, the scale bar on the TEM micrograph was used as the reference for setting the scale from pixel to nm using the line tool. Then a line was drawn for an imaginary diameter of a nanoparticle on the micrograph, and measure functionality under analysis was used to calculate its diameter in nm.

### 5.8 ELISA to Evaluate POEGMA Binding to Anti‐PEG Antibodies

mPEG (20 kDa, Creative PEGWorks) or POEGMA F at 200 µg mL^−1^ in PBS were coated onto MaxiSorp 96‐well plates (Nunc, ThermoScientific #439454) for 18 h at 4°C, followed by washing with PBS four times. Plates were blocked with 5% (w/v) skim milk powder in PBS overnight, followed by the addition of serially diluted (concentration range: 2–0.0325 µg/mL) anti‐PEG IgG (mouse monoclonal, Life Diagnostics #5D6‐3) or IgM (mAb, mouse, GenScript #A01795) in 5% skim milk in duplicate for 1 h at 22°C. Plates were then washed with 0.1% 3‐((3‐cholamidopropyl)dimethylammonio)‐1‐propanesulfonate (CHAPS, Sigma‐Aldrich, USA)/PBS buffer twice and PBS four times, before addition of a Biotin‐SP‐conjugated anti‐mouse IgM (Jackson Immunoresearch #115‐065‐075) or biotin‐conjugated goat anti‐mouse light chain IgG (Sigma #AP200B) at 0.2 µg/mL for 1 h at 22°C. Plates were washed as above, and then Streptavidin conjugated horseradish peroxidase (HRP Streptavidin, BioLegend #405210) was added at 0.2 µg/mL in 5% skim milk. The plates were washed again and then developed with 100 µL of pre‐mixed 3,3′,5,5′‐tetramethylbenzidine (TMB) liquid substrate (BioLegend # 421101) for 10 min at room temperature before stopping the reaction with the addition of 100 µL of Stop Solution (BioLegend #423001). The plates were read by quantifying absorbance at 450 nm on a Wallac 1420 Victor3 Plate Reader (Perkin Elmer). The background absorbance was detected by adding the antibodies to the non‐PEG or POEGMA‐coated wells, and by adding no antibodies to the coated wells, followed by the same ELISA procedure.

### 5.9 Hydrophobic Small Drug Encapsulation

To screen drug encapsulation in POEGMA nanoparticles, a 100 mM drug stock and a 10 mM polymer stock for POEGMA F were prepared in DMSO. Aliquots of the drug stock (1 µL) and the polymer stock (1 µL) were mixed with PBS (198 µL), and the mixture was incubated at room temperature for 10 min for the self‐assembly of POEGMA and drug encapsulation to occur. The solution was then centrifuged at 14 000 rcf for 5 min at room temperature to remove any precipitated but unencapsulated drug. The supernatant containing the POEGMA nanoparticles and drug mixture was then passed through 4–5 rounds of Amicon (10 k Mw cutoff) ultracentrifugation filters to remove free drug and isolate the POEGMA nanoparticles loaded with drug in PBS. The drug loading efficiency was evaluated by preparing standard curves from known concentrations of free drugs against their absorbance values measured on HPLC (Agilent 1100 LC system equipped with MassHunter Workstation software 10.1) in line with an Agilent EC C‐18 Poroshell column (4.6 × 50 mm, 2.7 µm particle size) for chromatographic separation at room temperature. The optimized mobile phase consisted of 30 mm sodium phosphate buffer, pH 5.6 (A) and acetonitrile (B) at a flow rate of 0.75 mL/min. Gradient separation for a 10 µL injection was achieved over a min run at 80% A and 20% B.

Paclitaxel encapsulation by (1) thin film hydration: Both the drug and the polymer stocks were prepared in chloroform (20 mg/mL). Paclitaxel and POEGMA F were mixed at a 10:1 molar ratio in chloroform, which was then dried on a rotary evaporator to create a thin film of the polymer and the drug. The thin film was hydrated with PBS at room temperature and left undisturbed overnight. (2) Buffer exchange method: Paclitaxel stock solution (20 mg/mL) was prepared in ethanol by intermittent vertexing and heating at 60°C. POEGMA stock (20 mg/mL) was prepared in 50% ethanol in water. The two stocks were mixed at a 10:1 drug to polymer molar ratio, and the formulation was dialyzed against PBS at room temperature. The buffer was replenished once after 24 h. Any excess paclitaxel that was not encapsulated in the polymer was removed from all the formulations by centrifugation at 14 000 rcf for 5 min at room temperature.

For large scale fabrication of Dox‐loaded POEGMA nanoparticles, a 500 µM stock Dox solution in PBS was used to resuspend the polymer at a Dox to polymer molar ratio of 10:1 at 4°C. After ensuring dissolution of the polymer, the solution was incubated at room temperature —a temperature above the CMT but below the *T*_t_ of the polymer—for self‐assembly of POEGMA nanoparticles followed by drug encapsulation. The solution was then centrifuged at 8000 rcf for 5 min at room temperature to remove any precipitated but unencapsulated Dox. The supernatant containing the polymer nanoparticles and Dox mixture was passed through several rounds of Amicon (10 k Mw cutoff) ultracentrifugation filters to remove free Dox and isolate Dox‐loaded POEGMA nanoparticles in PBS. With each round of ultracentrifugation filtration, the absorbance (*λ *= 480 nm) of the flowthrough was measured before discarding it, and the sample was diluted with PBS. This process was repeated until the Dox absorbance in the flow‐through was zero. The Dox encapsulated nanoparticles were tested for purity by running them on an agarose gel toward the negative terminal at 130 V for 30 min. The fluorescence of Dox was used to image the location of Dox in the gel. Any significant amount of free Dox in the sample was measured by its migration in the gel, while the encapsulated Dox stayed in the well, along with the polymer at the loading site. The encapsulation efficiency of Dox in POEGMA nanoparticles was quantified by a fluorescence‐based assay. Briefly, the Dox‐loaded nanoparticles were diluted 20x with acidified isopropanol (75 mM HCl, 10% H2O, 90% isopropanol) to disrupt the self‐assembly of nanoparticles, and the fluorescence of Dox was measured. A serial dilution series of known Dox solution was also generated in the same solvent system, and the measured fluorescence of Dox against its known concentration was plotted to obtain a linear calibration, which was then used to determine the Dox concentration in unknown samples.

### 5.10 Doxorubicin Release Assay

Phosphate–citrate buffer systems were prepared to model three biologically relevant environments: physiological pH (7.4), tumor microenvironment pH (6.5–7.0), and late endosome/lysosomal pH (~M5.0). Dox‐loaded POEGMA nanoparticles and free Dox (control) were placed into dialysis cassettes (MWCO 3.5 kDa, 3 mL capacity; Thermo Scientific #66330). Each cassette was submerged in 150 mL of the designated buffer and incubated at 37°C with gentle agitation for 168 h. Aliquots (50 µL) were collected from within the dialysis cassette at predetermined time points (0, 1, 2, 4, 8, 24, 48, 72, 120, 144, and 168 h), and the fluorescence signal was quantified using a microplate reader (λ_ex = 485 nm, λ_em = 590 nm). Dox concentrations were determined from standard calibration curves prepared in the corresponding buffer. Release profiles were generated by normalizing the remaining Dox within the cassette to the initial loading amount.

### 5.11 Biological Safety Assay

The biocompatibility of POEGMA was evaluated using a representative diblock polymer, POEGMA ‘F’ in two murine colorectal adenocarcinoma cell lines, MC38 and CT26. Cells were seeded at a density of 5 × 10^3^ cells per well by plating 90 µL of cell suspension into 96‐well plates 24 h before treatment. Serial dilutions of POEGMA (0–1000 µM) were prepared in culture medium, and 30 µL of each dilution was added to the wells (n = 3 per condition). Cells were incubated with polymer for 72 h at 37°C and 5% CO_2_. Cell viability was quantified using the CellTiter 96 AQueous MTS assay (Promega) as per manufacturer instructions. Briefly, 20 µL of the MTS reagent was added to each well and incubated for 1 h before measuring absorbance at 490 nm using a microplate reader. Blank wells (medium only) and wells treated with PBS served as 0% and 100% viability controls, respectively. The assay measures the enzymatic reduction of the tetrazolium reagent by metabolically active cells, enabling quantification of cell viability following treatment.

### 5.12 In Vitro Potency of Dox ‐Loaded Nanoparticles by MTS Assay

The efficacy of free Dox vs encapsulated Dox was investigated in C26 (RRID:CVCL_7254) murine colon adenocarcinoma cell line purchased from ATCC, and MC38 (UNSPSC Code: 41106514; RRID:CVCL_B288) murine colon adenocarcinoma cell line purchased from Sigma‐Aldrich. The cell lines were free of mycoplasma and were maintained in culture as per manufacturer's instructions. A 90 µL of cell suspension—corresponding to 5 × 10^3^ cells/well— were plated in a 96‐well format a day before the assay. Cells were treated with dilutions of the drug (free Dox or encapsulated Dox) in a volume of 30 µL/well (n = 3) and were incubated for 72 h. Blank wells and wells treated with PBS were defined as 0 and 100% viability, respectively. 20 µL of CellTiter 96 AQueous (Promega) reagent was added to each well and incubated for 1 h before measurement of the absorbance at 490 nm. The assay measures the reduction of tetrazolium reagent by metabolically active cells to determine cell viability after treatment. The data were analyzed by plotting Dox concentration vs absorbance, and a four‐parameter logistic model was fitted in GraphPad Prism to calculate the IC_50_ of the drug.

### 5.13 Uptake by Confocal Fluorescence Microscopy and Flow Cytometry

C26 and MC38 cells were seeded in an 8‐well chambered slide (ibidi cat# 80806) at a density of 20 × 10^3^ cells/well in 180 µL media and incubated overnight. Free Dox (20 µL) or Dox encapsulated in POEGMA nanoparticles at the equivalent Dox concentration were added to each well (n = 3) and incubated for 24 h. Cells were washed three times with 300 µL of PBS, and the nuclei were stained with Hoechst 33342 (Thermo Scientific, cat# 62249) according to the manufacturer's instructions. Imaging was performed using a Leica Stellaris confocal microscope with a 63x oil immersion objective, 405 nm laser (Hoechst), and 488 nm laser (Doxorubicin). Images were processed using the Leica LasX software. To quantify the uptake of doxorubicin by flow cytometry, cells were processed similarly. Briefly, after treatment with free Dox or Dox encapsulated in nanoparticles, cells were washed with PBS twice and then trypsinized and resuspended in 200 µL of complete media. The cell suspension was transferred to a 96‐well round bottom plate (Falcon cat# 351177), and cells were analyzed on a Beckman Coulter CytoFLEX flow cytometer. Untreated cells were used as a negative control. Data was analyzed using FlowJo and plotted in GraphPad Prism.

### 5.14 In Viv*o* Experiments

The animal studies were conducted under protocol A242‐23‐12 using procedures approved by the Duke Institutional Animal Care and Use Committee (IACUC). Duke University's Division of Laboratory Animal Resources (DLAR) maintains compliance with Duke's IACUC and its regulatory accreditation requirements. Duke's Animal Care and Use program is fully accredited by the Association for the Assessment and Accreditation of Laboratory Animal Care, International (AAALAC). Furthermore, Duke is registered as a research facility with the US Department of Agriculture (USDA) in accordance with the Animal Welfare Act and holds a Category I Assurance with the Public Health Service through the NIH's Office of Laboratory Animal Welfare (OLAW). All animal research at Duke University is conducted accordingly and upholds the standards put forth by these organizations.

### 5.15 Pharmacokinetics

To determine if encapsulation of Dox in POEGMA nanoparticles system alters its pharmacokinetics, we injected BALB/c mice (The Jackson Laboratories) with 4 mg/kg free Dox or encapsulated Dox (in POEGMA ‘F’) via the tail vein. The injection concentrations were prepared at 600 µm so that each mouse would be injected with a small volume. At pre‐determined time points (45 s, 10 m, 30 m, 2 h, 6 h, 10 h), 10 µL of blood was collected into 90 µL of 1000 U/mL heparin in PBS and kept on ice. Blood was centrifuged at 5000 g for 10 min at 4°C, and the plasma was then transferred to new tubes and stored at −80°C until further processing. The plasma was thawed, and a 30 µL aliquot was added to an Eppendorf tube containing 270 µL of acidified isopropanol (75 mM HCl, 10% H2O, 90% isopropanol). Dox standards were also prepared in acidified isopropanol to a final concentration range of 1 µm to 1.6 nm using five‐fold serial dilutions. The standards and samples were then incubated overnight at 4°C to ensure full release of all encapsulated Dox. After overnight incubation, the samples and standards were centrifuged at 5000 g for 5 min at 4°C. 125 µL of the supernatant was pipetted into a black, clear bottom 96‐well plate in duplicates, and the Dox fluorescence was quantified on a Wallac 1420 Victor3 Plate Reader (Perkin Elmer). Standard curves for free Dox and encapsulated Dox were very similar and were used to quantify the Dox concentration in the blood at each time point. The half‐life for free and encapsulated Dox was calculated by fitting a one‐phase exponential decay equation to data points in the elimination phase using Prism 7 software.

### 5.16 Tumor Regression Study

5‐week‐old female balb/c mice were obtained from the Jackson Laboratory and C26 tumor model was grafted by *s.c*. injection of 4 × 10^5^ C26 cells in 30 µL MEM into the right flank of mice. Tumors were allowed to grow for 10–14 days until the tumor size reached 75–100 mm^3^, at which time, the treatments were delivered via the tail vein at a Dox equivalent dose of 10 mg/kg. Tumor size (tumor volume (mm^3^) = length × width^2^ × π/6) and body weight were monitored daily. Mice with a tumor volume greater than 2000 mm^3^ or body weight loss greater than 15% were euthanized. The data was plotted as the change in tumor volume with time and survival curves after treatment. The Kaplan‐Meier survival curves were compared with a log‐rank test, whereas tumor regression curves were compared by a two‐way ANOVA followed by Tukey‐Kramer (Tukey's) post‐hoc test. All statistical analyses were performed using GraphPad Prism v.7.0 software.

### 5.17 Statistical Analysis

All quantitative data are presented as mean ± standard error (SEM) unless otherwise noted. Statistical analyses were performed using GraphPad Prism. Comparisons between two groups were conducted using unpaired two‐tailed Student's *t*‐tests. For comparisons involving more than two groups, one‐way or two‐way ANOVA (as appropriate) followed by Tukey's post‐hoc test was used to assess significance. Linear regression was applied for calibration curves used in drug quantification. A *p* value < 0.05 was considered statistically significant.

## Author Contributions

P.S. and A.C. conceived and designed the research. P.S., B.E.S., Y.S., S.D., J.T., Y.Y.M., C.S.P., S.S., X.L., and L.F. performed the experiments. M.R.N., S.Y.K., J.J.M., M.L.B., and D.R. contributed to experimental design. P.S. and A.C. interpreted the results and wrote the manuscript. All authors participated in the discussion of the data and commented on the manuscript.

## Conflicts of Interest

P.S., B.S., and A.C. have international patent application pending titled “BLOCK COPOLYMER NANOPARTICLES FOR SUSTAINED DRUG DELIVERY AND METHODS OF MAKING AND USING SAME” (Application No. PCT/US2024/050476). The technology described herein has been licensed by Veil Therapeutics from Duke University, and P.S., S.S., and A.C. have a financial interest in Veil Therapeutics.

## Supporting information




**Supporting File**: advs73894‐sup‐0001‐SuppMat.docx.

## Data Availability

The data that support the findings of this study are available from the corresponding author upon reasonable request.
