# Supplementary material for: All‐PEG‐Like Block Copolymers Self‐Assemble into Stealth Nanocarriers for Drug Delivery
Source: Adv Sci (Weinh). 2026 Jan 18;13(17):e17048. doi: 10.1002/advs.202517048 (PMC13042870; doi:10.1002/advs.202517048)
Supplement: Supplementary file 1 — Supporting File: advs73894‐sup‐0001‐SuppMat.docx. [file ADVS-13-e17048-s001.docx]

**All-PEG-like Block Copolymers Self-Assemble into Stealth Nanocarriers for Drug Delivery**

Parul Sirohi^1^, Brooke E. Silverstein^1^, Yulia Shmidov^1^, Sonal Deshpande^1^, Joy Tong^1^, Yvonne Y. Ma^1^, Chinmay S. Potnis^1^, Soumen Saha^1^, Xinghai Li^1^, Max R. Ney^1^, Sarah Y. Kim^1^, Joshua J. Milligan^1^, Lixin Fan^2^, Matthew L. Becker^1,3,4^, Daniel Reker^1^, Ashutosh Chilkoti^1^

^1^Department of Biomedical Engineering, Duke University, Durham, North Carolina 27708, United States

^2^Basic Science Program, Frederick National Laboratory for Cancer Research, SAXS Facility of the National Cancer Institute, Frederick, MD 21702, USA

^3^Department of Chemistry, Duke University, Durham, North Carolina 27708, United States

^4^Thomas Lord Department of Mechanical Engineering and Material Science, Duke University, Durham, North Carolina 27708, United States

**Supporting Information**

**Supplementary Figure 1**: Physical characterization of diblock POEGMA ‘A’. **A**) NMR confirming purity and chemical structure of the polymer. **B**) LCST phase behavior of the polymer characterized by absorbance at 600 nm at various concentrations as a function of solution temperature. Heating curves are represented by solid lines and cooling curves are represented by dashed lines. A sharp increase in absorbance around 30 °C for 100 µM in the heating curve indicates the cloud point (T_t_) at this concentration for this polymer. **C-D**) DLS analysis revealed that the polymer is a unimer at all temperatures below its T_t_ at low (12.5 µM) and high concentration (200 µM).

**Supplementary Figure 2**: Physical characterization of diblock POEGMA ‘B’. **A**) NMR. **B**) LCST phase behavior of the polymer characterized by absorbance at 600 nm at various concentrations as a function of solution temperature. Heating curves are represented by solid lines and cooling curves are represented by dashed lines. A sharp increase in absorbance at 43 °C for all concentrations in the heating curve indicates the T_t_ for this polymer. **C-D**) DLS analysis revealed that the polymer is a unimer at temperatures below 32 °C (CMT at 200 µM) but exhibit self-assembly for temperatures in between its CMT and T_t_. **E**) 1D SAXS data at 37°C for polymer B at 5 mg/ml: Zero slope at low q indicates spherical nanoparticles. **F**) A Guinier plot (linear region of ln(*I*) vs. q^2^ at q~0) to calculate R_g_ of the self-assembled nanoparticles using the formula R_g_ = sqrt(-3 * slope), results in a R_g_ of 10.7 nm for polymer B at 37 °C. **G**) The sharp peak in the Kratky plot validates compact self-assembly.

**Supplementary Figure 3**: Physical characterization of diblock POEGMA ‘C’. **A**) NMR. **B**) LCST phase behavior of the polymer characterized by absorbance at 600 nm at various concentrations as a function of solution temperature. Heating curves are represented by solid lines and cooling curves are represented by dashed lines. This is a complex phase behavior exhibiting multiple transitions due to larger aggregate formations but the final increase in absorbance at 43 °C for all concentrations in the heating curve indicates the T_t_ for this polymer. **C-D**) DLS analysis revealed that the polymer is a unimer at temperatures below 26 °C but exhibit aggregation for temperatures in between its CMT and T_t_. **E**) The size dependence on scattering angle indicates non-spherical assembly or polydispersity in the aggregation. **F**) 1D SAXS data at 37°C for polymer C at 1, 2 and 5 mg/ml indicates large cylindrical assemblies. **G**) cryo-EM at 30 °C.


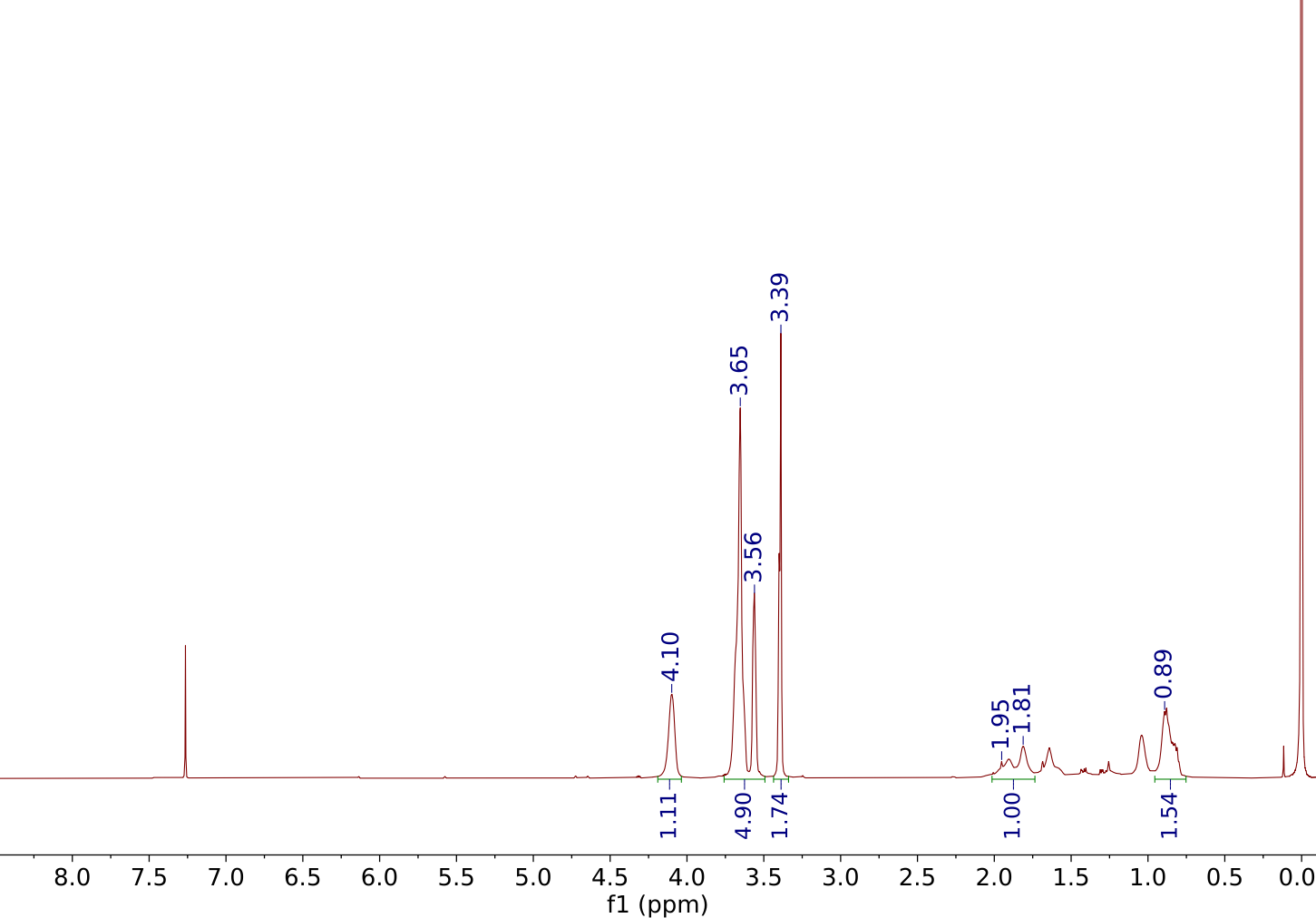


**Supplementary Figure 4**: NMR of diblock polymer ‘**D’**.

**Supplementary Figure 5**: Physical characterization of diblock POEGMA ‘E’. **A**) NMR. **B**) LCST phase behavior of the polymer characterized by absorbance at 600 nm at various concentrations as a function of solution temperature. Heating curves are represented by solid lines and cooling curves are represented by dashed lines. A sharp increase in absorbance around 45 °C for 100 µM in the heating curve indicates the T_t_ at this concentration. **C-D**) DLS analysis revealed that the polymer is a unimer at all temperatures below its T_t_ at both the low (12.5 µM) and high concentration (200 µM). **E**) 1D SAXS data at 37°C for polymer E at 5 mg/ml indicates small particles corresponding to unimers.

**Supplementary Figure 6**: **A**) NMR for polymer ‘F’. **B**) A defined peak in the kratky plot for polymer F at room temperature suggests a compact assembly.

**Supplementary Figure 7**: Physical characterization of diblock POEGMA ‘G’. **A**) NMR. **B**) LCST phase behavior of the polymer characterized by absorbance at 600 nm at various concentrations as a function of solution temperature. Heating curves are represented by solid lines and cooling curves are represented by dashed lines. A sharp increase in absorbance at 43.5 °C for all concentrations in the heating curve indicates the T_t_ for this polymer. **C-D**) DLS analysis revealed that the polymer is a nanoparticle of size around 56 nm in hydrodynamic diameter at all temperatures tested below its T_t_ at both the low (12.5 µM) and high concentration (200 µM).

**Supplementary Figure 8**: Physical characterization of diblock POEGMA ‘H’. **A**) NMR. **B**) LCST phase behavior of the polymer characterized by absorbance at 600 nm at various concentrations as a function of solution temperature. Heating curves are represented by solid lines and cooling curves are represented by dashed lines. A sharp increase in absorbance at 44.5 °C for all concentrations in the heating curve indicates the T_t_ for this polymer. **C-D**) DLS analysis revealed that there is some aggregation at low temperatures which is concentration dependent, and stable nanoparticle of size around 56 nm in hydrodynamic diameter between 30 - 40 °C.

**Supplementary Figure 9**: Physical characterization of diblock POEGMA ‘I’. **A**) NMR. **B**) LCST phase behavior of the polymer characterized by absorbance at 600 nm at various concentrations as a function of solution temperature. Heating curves are represented by solid lines and cooling curves are represented by dashed lines. A sharp increase in absorbance at 45 °C for all concentrations in the heating curve indicates the T_t_ for this polymer. **C-D**) DLS analysis revealed that the polymer is a nanoparticle of size around 25 nm in hydrodynamic diameter at all temperatures tested below its T_t_ at both the low (12.5 µM) and high concentration (200 µM). **E-F**) A polymer excluded-volume model provided the best fit for the 1-D SAXS data of polymer I at room temperature, which is typically used to describe porous, irregular, or partially collapsed polymer aggregates. This model resulted in an R_g_ of 11.34 nm—larger than the Guinier-derived R_g_—and a Porod exponent of 1.6, indicative of a semiflexible chain rather than a compact nanoparticle. The Kratky plot further supported this interpretation, displaying a broad, poorly defined peak consistent with an ill-organized or heterogeneous morphology.

**Supplementary Figure 10**: Physical characterization data showing that removal of the CTA group from the diblock POEGMA have no impact on the phase behavior and self-assembly of polymer F.

**Supplementary Figure 11**: Zeta potential measurement of polymer F in water at room temperature revealed a slight negative change as expected for a nanoparticle formed form a neutral polymer.

**Supplementary Figure 12**: Calibration curves used for quantifying drug encapsulation efficiency. **A–E**) Linear calibration curves generated for each drug using an HPLC-based quantification method. Absorbance peak area was plotted against known drug concentrations to obtain regression models. **F**) Calibration curve for doxorubicin generated using fluorescence measurements on a plate reader, establishing a linear relationship between Dox concentration and fluorescence intensity.

**Supplementary Table 1**: Drug loading efficiency and capacity.

| **Drug** | **Molecular weight** | **log P** | **% Encapsulation efficiency** | **% Loading capacity (w/w)** |
| --- | --- | --- | --- | --- |
| Fulvestrant | 606.78 | 8.9 | 69 | 0.6 |
| Lapatinib | 581 | 5.4 | 58 | 0.5 |
| Sorafenib | 464.82 | 4.12 | 76 | 0.7 |
| Paclitaxel | 853.9 | 3.5 | 90 | 10 |
| Erlotinib | 393.44 | 2.7 | 52 | 0.4 |
| Doxorubicin | 579.98 | 1.27 | 50 | 0.5 |

Drug encapsulation efficiency was calculated as the ratio of the amount of drug retained in the nanoparticles to the total starting drug amount. Loading capacity (w/w) is defined as the mass (in milligram) of drug encapsulated per milligram of polymer.

**Supplementary Figure 13**: Size distribution profiles of polymer ‘F’ nanoparticles before and after drug loading.

**Supplementary Figure 14**: Doxorubicin (Dox) release profiles from POEGMA nanoparticles under physiologically relevant conditions. **A**) free Dox controls, and **B**) Dox-loaded POEGMA nanoparticles were placed in dialysis cassette (MWCO 3.5 kDa) and dialyzed against four buffers representing key biological environments: physiological pH (7.4), tumor microenvironment (pH 6.5–7.0), and late endosomal/lysosomal (pH ~5.0) conditions. Samples were collected from inside the cassette over 168 hours at 37 °C and % Dox retention was quantified by fluorescence. Free Dox rapidly diffused out of the cassette within the first 10 hours across all conditions, whereas Dox encapsulated within POEGMA nanoparticles exhibited sustained retention for the full duration of the study, with accelerated release observed at lower pH.

**Supplementary Figure 15**: *In vitro* Cell viability after 72 h incubation with increasing concentrations of POEGMA ‘F’ (0–250 μM). Viability was assessed using a metabolic assay and normalized to untreated controls. Data is presented as mean ± SD (n = 3). POEGMA showed no detectable cytotoxicity in either cell line across all tested concentrations, demonstrating its high biocompatibility.

**Supplementary Figure 16**: *In vitro* potency of Dox in a MC38 colon carcinoma cell line after 24, 48 and 72 h incubation with the free drug or drug encapsulated in polymer ‘F’.

**Supplementary Figure 17**: *In vitro* potency of Paclitaxel in a PC3 prostate cancer cell line after 48 and 72 h incubation with the free drug or drug encapsulated in polymer **F**.

**Supplementary Figure 18**: Uptake of Dox by C26 after 24h of incubation with Dox equivalent dose of 3.5 μM by confocal imaging. Scale bar is 50 μm.

**Supplementary Figure 19**: Uptake of Dox by MC38 after 24h of incubation with Dox equivalent dose of 4 μM by confocal imaging. Scale bar is 20 μm.

**Supplementary Figure 20**: Uptake of Dox by MC38 after 24h of incubation with Dox equivalent dose of 1.7 or 3.5 μM by flow cytometry.

**Supplementary Figure 21**: Physiological stability of POEGMA nanoparticles. **A**) Size distribution of serum control. Size distribution of **B**) empty POEGMA ‘F’ nanoparticles, and **C**) nanoparticles loaded with Doxorubicin after 0 to 168 h of incubation in medium containing 20% serum at 37 °C. DLS measurements showed no appreciable change in particle size or dispersity over 168 h, indicating excellent serum stability. **D**) Agarose gel showing intact Dox encapsulation inside the POEGMA nanoparticle even after 168 h of serum incubation at 37 °C.

**Supplementary Figure 22**: Pharmacokinetics of Dox encapsulated in polymer ‘**F’** as compared to free Dox after intravenous administration of the drug at a Dox equivalent dose of 4 mg/kg (n =3). The Dox fluorescence signal could be detected up to 2 h for encapsulated Dox and for only 45 seconds in the case of free Dox. The drug elimination half-life (t_1/2_) was calculated by fitting a one phase decay model to the PK curve. The total drug exposure in plasma was calculated by area under the PK curve (AUC). Additionally, the plasma Dox concentration immediately 45 seconds after the injection indicated a significantly altered biodistribution of Dox.

**Supplementary Figure 23**: The longitudinal body weight measurements for all treatment groups during the Dox anti-tumor efficacy study.
